# Effectiveness of calcipotriol/betamethasone dipropionate aerosol foam in patients with small versus large plaque psoriasis in routine practice in South Korea

**DOI:** 10.1111/1346-8138.17253

**Published:** 2024-05-08

**Authors:** Seong Jin Jo, Chul‐Jong Park, Chul Hwan Bang, Ki‐Heon Jeong, Bong Seok Shin, Dong Hyun Kim, Hae Jun Song, Ju‐Hee Lee, YoungEun Kim, Sun Choi, Sang Woong Youn

**Affiliations:** ^1^ Department of Dermatology Seoul National University College of Medicine Seoul South Korea; ^2^ Department of Dermatology Seoul National University Hospital Seoul South Korea; ^3^ Department of Dermatology, Bucheon St. Mary's Hospital The Catholic University of Korea Bucheon South Korea; ^4^ Department of Dermatology, Seoul St. Mary's Hospital The Catholic University of Korea Seoul South Korea; ^5^ Department of Dermatology KyungHee University Medical Center Seoul South Korea; ^6^ Department of Dermatology Chosun University Hospital Gwangju South Korea; ^7^ Department of Dermatology CHA Bundang Medical Center, CHA University School of Medicine Seongnam South Korea; ^8^ Department of Dermatology Chungnam National University Sejong Hospital Sejong South Korea; ^9^ Department of Dermatology Severance Hospital Seoul South Korea; ^10^ LEO Pharma Limited Seoul South Korea; ^11^ Department of Dermatology Seoul National University College of Medicine Seongnam South Korea; ^12^ Department of Dermatology Seoul National University Bundang Hospital Seongnam South Korea

**Keywords:** betamethasone dipropionate, calcipotriol, fixed dose combination, Korean psoriasis, plaque size

## Abstract

Small plaque psoriasis is the typical form of chronic plaque psoriasis affecting adults in South Korea. The effectiveness of calcipotriol/betamethasone dipropionate (Cal/BD) aerosol foam for large and small psoriasis plaques has not previously been examined. We performed a post hoc analysis of a recent, 4‐week observational study of Cal/BD aerosol foam use in routine clinical practice in South Korea. Investigator Global Assessment response ([IGA] 0/1 at week 4), Patient Global Assessment response ([PaGA] 0/1 at week 4), change in Psoriasis Area and Severity Index (PASI), changes in psoriasis symptom scores, change in the Dermatology Life Quality Index (DLQI), and the proportion of patients achieving DLQI ≤5 were analyzed for patients with small (≤5 cm; *n* = 131) or large (>5 cm; *n* = 35) baseline plaque size. IGA response rates were similar for patients with small and large plaques (59.5% and 51.4% respectively). Similarly, there was no significant difference between the small and large groups in mean change in PASI (−2.20 vs −3.34), the proportions of patients with DLQI ≤5 (62.3% vs 54.3%) or PaGA 0/1 (29.2% vs 40.0%). Mean improvements in DLQI (−4.04 vs −6.20) and in psoriasis symptoms including itching (−1.50 vs −2.83), sleep loss (−0.67 vs −1.89), dryness (−1.57 vs −2.97), scaling (−1.21 vs −3.57), and redness (−1.17 vs −3.11) were greater in patients with large plaques than those with small plaques. Itching and DLQI differences were not statistically significant after adjustment for baseline characteristics. Stratification by body surface area affected eliminated statistically significant differences between the groups for most outcomes. In conclusion, this analysis suggests that Cal/BD aerosol foam is an effective, well‐accepted treatment for adult patients with the small plaques typical of chronic plaque psoriasis in South Korea, as well as for those with large plaques.

## 1 INTRODUCTION

Psoriasis is a chronic, immune‐mediated, inflammatory skin disease.^1^ Psoriasis vulgaris (plaque psoriasis), characterized by red, scaly plaques, accounts for 90% of cases.^1^ In Asian populations, a distinct psoriasis phenotype, “small plaque psoriasis”, has been reported.^2^, ^3^ Small plaque psoriasis is the typical form of chronic plaque psoriasis seen in adults in South Korea,^2^ where around 0.5% of the population is affected.^4^

Although data are sparse, several studies have suggested possible differences in the effectiveness of some psoriasis treatments between large and small plaques.^4^, ^5^, ^6^, ^7^ However, no recent studies have specifically assessed the effectiveness of topical therapies, which are used by most patients with psoriasis in South Korea, in the treatment of small psoriasis plaques.

One topical therapy that has been shown to be effective for plaque psoriasis in clinical trials and observational studies is calcipotriol/betamethasone dipropionate (Cal/BD) aerosol foam (Enstilum, LEO Pharma).^8^, ^9^, ^10^, ^11^ Data on Cal/BD aerosol foam treatment outcomes in Asian patients are limited. However, a recent observational study of Cal/BD aerosol foam use in routine clinical practice in South Korea found that patients showed improvements in lesions and health‐related quality of life after 4 weeks, with a high level of treatment satisfaction and good overall tolerability and safety.^12^ The patient population included patients with both small plaque psoriasis and those with large plaques.^12^ This enabled treatment outcomes according to plaque size to be compared.

The objectives of this post hoc analysis were to evaluate treatment outcomes and treatment satisfaction in South Korean patients with small versus large psoriasis plaques using Cal/BD aerosol foam in routine clinical practice.

## 2 METHODS

### 2.1 Study design and patients

Adults in South Korea receiving treatment with Cal/BD aerosol foam for psoriasis vulgaris were enrolled in a previously described, 4‐week, prospective, open‐label, non‐comparative, non‐interventional study.^12^

### 2.2 Post hoc analysis

Treatment effectiveness and patient‐reported outcomes (PROs) were analyzed for patients with small (≤5 cm) versus large (>5 cm) baseline plaque size; 5 cm has been used as a threshold in previous studies of small plaque psoriasis.^2^, ^3^, ^13^ Patients with both large and small plaques were categorized based on the mean plaque size of lesions with the most widespread distribution.

The outcomes analyzed were Investigator Global Assessment (IGA) response (0 or 1 at week 4), Patient Global Assessment (PaGA) response (0 or 1 at week 4), change in Psoriasis Area and Severity Index (PASI), changes in psoriasis symptom scores, change in Dermatology Life Quality Index (DLQI), and the proportion of patients achieving DLQI ≤5.

Statistical analyses were conducted adjusting for baseline differences (including sex, age, previous treatment, and body surface area [BSA] affected) between subgroups. Additionally, psoriasis symptoms were assessed according to BSA affected as well as plaque size. All analyses were descriptive and exploratory.

## 3 RESULTS

### 3.1 Clinical effectiveness

Baseline demographics were generally comparable between patients with small (*n* = 131) and large (*n* = 35) plaques (Table 1). However, compared with patients with small plaques, those with large plaques were significantly less likely to have received previous treatment for psoriasis; were significantly more likely to have diabetes mellitus; had a statistically significantly larger mean BSA affected; and had statistically significantly higher mean PASI, IGA, PaGA, itching, sleep loss, scaling, and redness scores.

**TABLE 1:** Baseline characteristics.

Characteristic	Plaque size		Total (*N* = 166)	*p* value
	≤5 cm (*n* = 131)	>5 cm (*n* = 35)		
Male sex, *n* (%)	76 (58.0)	24 (68.6)	100 (60.2)	0.2570^a^
Age, years				
Mean ± SD	47.2 ± 15.5	45.4 ± 13.2	46.8 ± 15.0	0.5234^b^
Median (range)	48 (20–81)	42 (23–76)	47 (20–81)	
Duration of psoriasis, years				
Mean ± SD	11.0 ± 12.5	9.2 ± 8.1	10.6 ± 11.7	0.8688^b^
Median (range)	6.9 (0.0–55.0)	6.9 (0.0–26.0)	6.9 (0.0–55.0)	
BSA affected, %				
Mean ± SD	5.5 ± 5.9	10.2 ± 8.5	6.5 ± 6.8	**0.0011** ^b^
Median (range)	3 (0.3–30.0)	9 (0.3–30.0)	4 (0.3–30.0)	
PASI				
Mean ± SD	5.1 ± 3.7	8.1 ± 5.8	5.7 ± 4.4	**0.0119** ^b^
Median (range)	4.0 (0.3–27.1)	5.2 (0.9–20.0)	4.2 (0.3–27.1)	
IGA				
Mean ± SD	2.3 ± 0.5	2.6 ± 0.8	2.3 ± 0.6	**0.0152** ^b^
Median (range)	2 (2–4)	2 (2–4)	2 (2–4)	
IGA, *n* (%)				
Mild (IGA 2)	99 (75.6)	20 (57.1)	119 (71.7)	**0.0049** ^a^
Moderate (IGA 3)	28 (21.4)	9 (25.7)	37 (22.3)	
Severe (IGA 4)	4 (3.1)	6 (17.1)	10 (6.0)	
IGA ≥2, *n* (%)	131 (100.0)	35 (100.0)	166 (100.0)	**–**
PaGA				
Mean ± SD	2.6 ± 0.7	2.9 ± 0.8	2.7 ± 0.8	**0.0356** ^b^
Median (range)	2 (1–4)	3 (2–4)	3 (1–4)	
PaGA, *n* (%)				
Almost clear (PaGA 1)	1 (0.8)	0 (0.0)	1 (0.6)	0.0717^c^
Mild (PaGA 2)	66 (50.4)	13 (37.1)	79 (47.6)	
Moderate (PaGA 3)	47 (35.9)	11 (31.4)	58 (34.9)	
Severe (PaGA 4)	17 (13.0)	11 (31.4)	28 (16.9)	
Psoriasis severity (PaGA) ≥ 2, *n* (%)	130 (99.2)	35 (100.0)	165 (99.4)	1.0000^c^
DLQI				
Mean ± SD	10.0 ± 6.7	12.4 ± 7.0	10.5 ± 6.8	0.0555^b^
Median (range)	9.0 (0.0–30.0)	12.0 (1.0–29.0)	9.5 (0.0–30.0)	
DLQI ≤5, *n* (%)	39 (29.8%)	6 (17.1%)	45 (27.1%)	0.1354^a^
Psoriasis symptoms (Itching)				
Mean ± SD	4.4 ± 2.8	5.6 ± 2.6	4.6 ± 2.8	**0.0187** ^b^
Median (range)	4 (1–10)	6 (1–10)	4 (1–10)	
Psoriasis symptoms (Sleep loss)				
Mean ± SD	2.6 ± 2.3	3.9 ± 2.9	2.9 ± 2.5	**0.0148** ^b^
Median (range)	2 (1–10)	4 (1–10)	2 (1–10)	
Psoriasis symptoms (Dryness)				
Mean ± SD	6.5 ± 2.8	7.1 ± 2.3	6.6 ± 2.7	0.2776^b^
Median (range)	7 (1–10)	8 (3–10)	7 (1–10)	
Psoriasis symptoms (Scaling)				
Mean ± SD	4.7 ± 3.1	6.3 ± 2.9	5.0 ± 3.1	**0.0048** ^b^
Median (range)	4 (1–10)	7 (1–10)	5 (1–10)	
Psoriasis symptoms (Redness)				
Mean ± SD	5.4 ± 3.1	7.1 ± 2.6	5.8 ± 3.1	**0.0034** ^b^
Median (range)	5 (1–10)	7 (1–10)	6 (1–10)	
Comorbidities, *n* (%)				
Any comorbidity	65 (49.6)	17 (48.6)	82 (49.4)	0.9124^a^
Hypertension	23 (17.6)	4 (11.4)	27 (16.3)	0.3828^a^
Diabetes mellitus	7 (5.3)	6 (17.1)	13 (7.8)	**0.0321** ^c^
Hyperlipidemia	10 (7.6)	2 (5.7)	12 (7.2)	1.0000^c^
Previous treatment				
Previous treatment for psoriasis, *n* (%)	88 (67.2)	17 (48.6)	105 (63.3)	**0.0426** ^a^
Previous treatment with topical drug, *n* (%)	82 (62.6)	13 (37.1)	95 (57.2)	**0.0069** ^a^
Previous treatment with systemic therapy, *n* (%)	35 (26.7)	1 (2.9)	36 (21.7)	**0.0023** ^a^

*Note*: Bold values indicate statistical significance.

Abbreviations: BSA, body surface area; DLQI, Dermatology Life Quality Index; IGA, Investigator Global Assessment; PaGA, Patient Global Assessment; PASI, Psoriasis Area Severity Index; SD, standard deviation.

^a^ Chi‐squared test.

^b^ Wilcoxon rank‐sum test.

^c^ Fisher's exact test.

**FIGURE 1:**
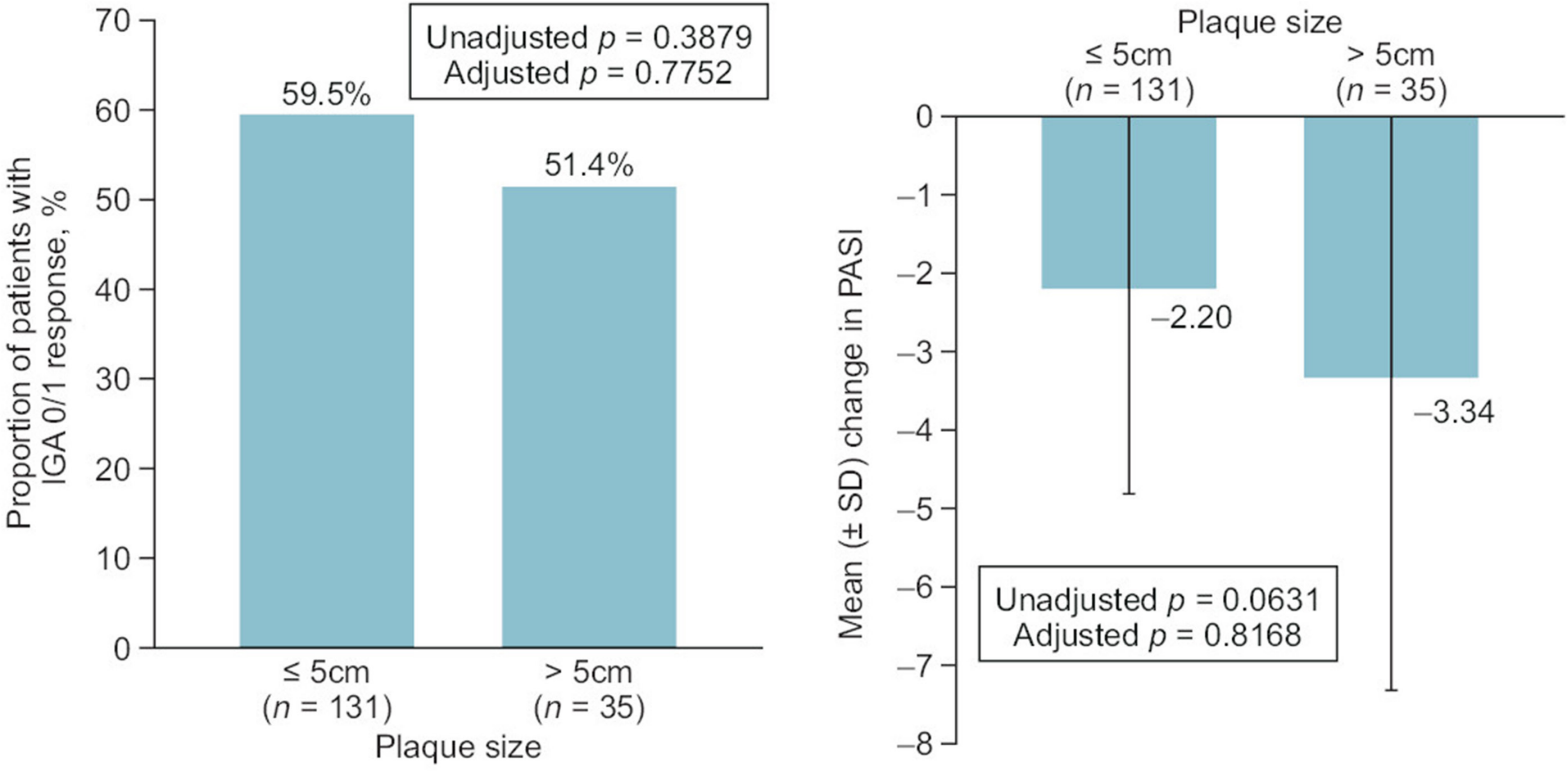
Proportions of patients with an Investigator Global Assessment (IGA) response and mean change in Psoriasis Area and Severity Index (PASI) at week 4 (*N* = 166). *p*‐values were calculated using the chi‐squared test (IGA response) or Wilcoxon rank‐sum test (mean change in PASI). Analyses were adjusted for sex, age, previous treatment with topical drug, previous treatment with systemic therapy and body surface area affected. SD, standard deviation.

Investigator Global Assessment response rates were similar for patients with small and large plaques with a majority in both groups achieving IGA 0/1 at week 4 (Figure 1). Similarly, there was no significant difference between the groups in mean change in PASI (Figure 1).

### 3.2 Patient‐reported outcomes

The proportion of patients achieving DLQI ≤5 was similar in the two groups, as was the PaGA response rate (Table 2).

**TABLE 2:** Patient‐reported outcomes.

Outcomes at week 4	Plaque size		Total (*N* = 165)	*p* value	*p* value^a^
	≤5 cm (*n* = 130)	>5 cm (*n* = 35)			
PaGA					
Patients with PaGA 0/1, *n* (%)	38 (29.2)	14 (40.0)	52 (31.5)	0.2235^b^	0.1259
DLQI					
Patients with DLQI ≤5, *n* (%)	81 (62.3)	19 (54.3)	100 (60.6)	0.3886^b^	0.9203
Change from baseline, mean ± SD	−4.04 ± 4.86	−6.20 ± 5.91	−4.50 ± 5.16	**0.0226** ^c^	0.0706
Score at endpoint, mean ± SD	5.89 ± 6.23	6.23 ± 5.61	5.96 ± 6.09	0.6063^c^	0.6432
Psoriasis symptoms					
Change from baseline, mean ± SD					
Itching	−1.50 ± 2.39	−2.83 ± 2.77	−1.78 ± 2.52	**0.0024** ^c^	0.0554
Sleep loss	−0.67 ± 2.12	−1.89 ± 3.39	−0.93 ± 2.49	**0.0109** ^c^	**0.0486**
Dryness	−1.57 ± 3.00	−2.97 ± 2.64	−1.87 ± 2.97	**0.0188** ^c^	**0.0491**
Scaling	−1.21 ± 2.94	−3.57 ± 3.42	−1.71 ± 3.19	**<0.0001** ^c^	**0.0033**
Redness	−1.17 ± 2.76	−3.11 ± 2.58	−1.58 ± 2.83	**0.0004** ^c^	**0.0013**
Score at endpoint, mean ± SD					
Itching	2.87 ± 2.00	2.74 ± 2.19	2.84 ± 2.03	0.5137^c^	0.6570
Sleep loss	1.97 ± 1.83	2.03 ± 2.01	1.98 ± 1.87	0.8088^c^	0.9278
Dryness	4.88 ± 2.65	4.14 ± 2.63	4.72 ± 2.65	0.1114^c^	**0.0419**
Scaling	3.42 ± 2.67	2.77 ± 2.52	3.28 ± 2.65	0.1459^c^	0.0933
Redness	4.19 ± 2.78	4.03 ± 2.62	4.16 ± 2.74	0.8675^c^	0.2578

*Note*: Bold values indicate statistical significance.

Abbreviations: BSA, body surface area; DLQI, Dermatology Life Quality Index; PaGA, Patient Global Assessment; SD, standard deviation.

^a^ Analyses were adjusted for sex, age, previous treatment with topical drug, previous treatment with systemic therapy and BSA affected.

^b^ Chi‐squared test.

^c^ Wilcoxon rank‐sum test.

Mean improvements in psoriasis symptoms and DLQI were greater in patients with large plaques, compared with those with small plaques, though mean scores were similar between the groups (Table 2). Differences in the improvement in itching and DLQI were not statistically significant after adjustment for baseline characteristics. When the results were stratified by BSA affected, the statistical significance of differences in most PROs was eliminated (except scaling and redness in one and two BSA categories, respectively) (Table S1).

### 3.3 Treatment satisfaction

Treatment satisfaction was high in both groups with patients with large plaques reporting significantly higher satisfaction with the ease of use of Cal/BD aerosol foam than those with small plaques (Table S2).

## 4 DISCUSSION

Over 4 weeks of real‐world use, Cal/BD aerosol foam improved lesion severity and PROs in South Korean patients with psoriasis, regardless of plaque size.

As expected in a South Korean population, most included patients had small plaque psoriasis. No significant differences were found between the groups in the effect of Cal/BD aerosol foam on investigator‐assessed measures of psoriasis severity or, after adjustment for baseline characteristics, in mean DLQI improvement. There was no difference between groups in the proportion of patients achieving DLQI ≤5, which corresponds to psoriasis having no effect or a small effect on patients' lives,^14^ after treatment with Cal/BD aerosol foam.

In unadjusted analyses, some differences were seen in the reported improvements in psoriasis symptoms, though mean scores at week 4 were similar. After adjustment for baseline characteristics and stratification by BSA affected, most differences were not statistically significant, suggesting generally similar treatment effectiveness across the groups.

Patients reported a generally high level of overall satisfaction and satisfaction with the effectiveness of Cal/BD aerosol foam, regardless of plaque size. Patients with large plaques found Cal/BD aerosol foam to be more convenient, compared with those with small plaques. This may reflect the Cal/BD foam aerosol administration method, which distributes the foam widely, allowing quick and easy coverage.^12^, ^15^

Few previous studies have compared the treatment of small and large psoriasis plaques with inconsistent differences in effectiveness according to plaque size reported for several therapies.^6^, ^7^, ^13^ Causes of these differences are unclear but may relate to differences in plaque thickness and disease pathogenesis between plaques of different sizes.^5^ It is notable, therefore, that in the present study there were no significant differences in the overall effectiveness of Cal/BD aerosol foam between the groups.

This study has some limitations. First, there were differences in baseline characteristics between the groups including the proportion of patients who had previously received topical and systemic treatments for psoriasis. This is likely to reflect the tertiary setting of the study with small plaque psoriasis possibly more likely than large plaque psoriasis to be treated initially in primary or secondary care. The statistical analysis was adjusted for these differences. In addition, as all patients were receiving Cal/BD aerosol foam in routine clinical practice, these differences do not affect the relevance of the study findings to South Korean patients. Second, some mean baseline symptom scores were higher in the large plaque group than in the small plaque group; this may explain some of the observed differences in symptom improvement after treatment. Third, comparisons between small and large plaques stratified by BSA may lack statistical power as the number of patients in some subgroups was low. However, no trend was observed according to plaque size, and the limited sample size should not affect the overall conclusions. Fourth, as in the original observational study, the potential for selection bias exists; this is likely to apply to both patient groups and should not affect the interpretation of the results.

In conclusion, this analysis suggests that Cal/BD aerosol foam is an effective, well‐accepted treatment for adult patients with the small plaques typical of chronic plaque psoriasis in South Korea, as well as for those with large plaques.

## CONFLICT OF INTEREST STATEMENT

Seong Jin Jo has served as an investigator in clinical trials sponsored by AbbVie, Boehringer Ingelheim, Bristol Myers Squibb, Celltrion, GC Cell, Janssen, LEO Pharma, Novartis, Pfizer, and UCB, and has received consultancy fees from AbbVie, Boehringer Ingelheim, GC Cell, Janssen, Kolon Pharma, LEO Pharma, Lilly, and Novartis and fees for speaking from AbbVie, Janssen, LEO Pharma, Lilly, Novartis and Sanofi. Chul Hwan Bang has received funds for research from Janssen, consultancy fees or commissioned fee‐paid work from Janssen and Novartis and fees for speaking from AbbVie, Janssen, Lilly and Novartis. Hae Jun Song has received funds for research from LEO Pharma. Ju‐Hee Lee has received funds for research from LEO Pharma. Young Eun Kim and Sun Choi are employees of LEO Pharma. Sang Woong Youn has served as an investigator in clinical trials sponsored by LEO Pharma, AbbVie, Janssen, Novartis, Boehringer Ingelheim, BMS, Eli Lilly, Kyowa Kirin, Celltrion, Samsung Bioepis, and UCB and has received fees for speaking from AbbVie, Janssen, Novartis, Boehringer Ingelheim, Eli Lilly, LEO Pharma and Pfizer. All other authors have no conflicts of interest to declare.

## ETHICS STATEMENT

The original study^12^ was conducted in accordance with the principles of the Declaration of Helsinki and approved by the local institutional review boards of all participating centers. All patients provided informed consent.

## Supporting information


Table S1.
Table S2.
