# Supplementary material for: Effectiveness of calcipotriol/betamethasone dipropionate aerosol foam in patients with small versus large plaque psoriasis in routine practice in South Korea
Source: J Dermatol. 2024 May 8;51(7):1010–6. doi: 10.1111/1346-8138.17253 (PMC11483922; doi:10.1111/1346-8138.17253)
Supplement: Supplementary file 1 — Table S1. Table S2. [file JDE-51--s001.docx]

SUPPLEMENTARY TABLE 1. Change in psoriasis symptoms by BSA affected

| Change in psoriasis symptoms, mean ± SD (n) | Plaque size | | | Total (*N* = 165) | | *P*-value | *P*-value^†^ |
| --- | --- | --- | --- | --- | --- | --- | --- |
|  | ≤5 cm (*n* = 130) | | >5 cm (*n* = 35) |  |  |  |  |
| Itching | | | | | | | |
| BSA <3% | −1.55 ± 2.19 (n = 42) | −3.14 ± 2.61 (n = 7) | | | −1.78 ± 2.29 (*n* = 49) | 0.1233^‡^ | 0.1751 |
| BSA 3–10% | −1.31 ± 2.47 (n = 64) | −2.83 ± 2.52 (n = 12) | | | −1.55 ± 2.52 (n = 76) | 0.0238^‡^ | 0.3836 |
| BSA ≥10% | −1.92 ± 2.54 (n = 24) | −2.69 ± 3.16 (n = 16) | | | −2.23 ± 2.79 (n = 40) | 0.3034^‡^ | 0.1953 |
| Sleep loss | | | | | | | |
| BSA <3% | −0.74 ± 2.41 (n = 42) | −2.14 ± 1.57 (n = 7) | | | −0.94 ± 2.35 (*n* = 49) | 0.0508^‡^ | 0.2653 |
| BSA 3–10% | −0.61 ± 1.77 (n = 64) | −2.17 ± 2.72 (n = 12) | | | −0.86 ± 2.01 (n = 76) | 0.0272^‡^ | 0.0571 |
| BSA ≥10 | −0.71 ± 2.51 (n = 24) | −1.56 ± 4.41 (n = 16) | | | −1.05 ± 3.37 (n = 40) | 0.6433^‡^ | 0.2838 |
| Dryness | | | | | | | |
| BSA <3% | −1.52 ± 2.81 (n = 42) | −3.14 ± 2.97 (n = 7) | | | −1.76 ± 2.86 (*n* = 49) | 0.1682^§^ | 0.3208 |
| BSA 3–10% | −1.50 ± 3.17 (n = 64) | −3.75 ± 2.34 (n = 12) | | | −1.86 ± 3.15 (n = 76) | 0.0234^‡^ | 0.1860 |
| BSA ≥10% | −1.83 ± 2.93 (n = 24) | −2.31 ± 2.70 (n = 16) | | | −2.03 ± 2.81 (n = 40) | 0.6964^‡^ | 0.1653 |
| Scaling | | | | | | | |
| BSA <3% | −1.24 ± 3.18 (n = 42) | −3.86 ± 2.48 (n = 7) | | | −1.61 ± 3.21 (*n* = 49) | 0.0442^§^ | 0.1627 |
| BSA 3–10% | −0.77 ± 2.59 (n = 64) | −4.42 ± 2.47 (n = 12) | | | −1.34 ± 2.88 (n = 76) | 0.0001^‡^ | 0.0030 |
| BSA ≥10% | −2.33 ± 3.17 (n = 24) | −2.81 ± 4.29 (n = 16) | | | −2.53 ± 3.62 (n = 40) | 0.6869^c^ | 0.7869 |
| Redness | | | | | | | |
| BSA <3% | −0.67 ± 3.26 (n = 42) | −3.86 ± 2.34 (n = 7) | | | −1.12 ± 3.32 (*n* = 49) | 0.0201^‡^ | 0.0146 |
| BSA 3–10% | −1.41 ± 2.52 (n = 64) | −3.33 ± 2.64 (n = 12) | | | −1.71 ± 2.62 (n = 76) | 0.0204^‡^ | 0.0285 |
| BSA ≥10% | −1.42 ± 2.38 (n = 24) | −2.63 ± 2.68 (n = 16) | | | −1.90 ± 2.54 (n = 40) | 0.2264^‡^ | 0.2310 |

^†^ Analyses were adjusted for gender, age, previous treatment with topical drug, previous treatment with systemic therapy and BSA affected.

^‡^ Wilcoxon rank-sum test.

^§^ Student's t-test.

BSA, body surface area; SD, standard deviation.

SUPPLEMENTARY TABLE 2. Treatment satisfaction

| Satisfaction with treatment, *n* (%) | Plaque size | | Total (*N* = 165) | *P*-value^†^ | *P*-value^‡^ |
| --- | --- | --- | --- | --- | --- |
|  | ≤5 cm (*n* = 130) | >5 cm (*n* = 35) |  |  |  |
| Overall satisfaction | | | | | |
| Satisfied | 93 (71.5%) | 29 (82.9%) | 122 (73.9%) | 0.3694 | 0.1508 |
| Neutral | 28 (21.5%) | 4 (11.4%) | 32 (19.4%) |  |  |
| Not Satisfied | 9 (6.9%) | 2 (5.7%) | 11 (6.7%) |  |  |
| Effectiveness | | | | | |
| Satisfied | 97 (74.6%) | 30 (85.7%) | 127 (77.0%) | 0.3725 | 0.2604 |
| Neutral | 22 (16.9%) | 3 (8.6%) | 25 (15.2%) |  |  |
| Not Satisfied | 11 (8.5%) | 2 (5.7%) | 13 (7.9%) |  |  |
| Ease of use | | | | | |
| Satisfied | 71 (54.6%) | 28 (80.0%) | 99 (60.0%) | 0.0240 | 0.0115 |
| Neutral | 30 (23.1%) | 4 (11.4%) | 34 (20.6%) |  |  |
| Not Satisfied | 29 (22.3%) | 3 (8.6%) | 32 (19.4%) |  |  |

^†^ *p* values were calculated using the Chi-square test.

^‡^ Analyses were adjusted for gender, age, previous treatment with topical drug, previous treatment with systemic therapy and BSA affected.

BSA, body surface area.
